# The Synthesis, Characterization, and Assessment of Antibacterial Properties of an Orthodontic Adhesive Containing Cerium-Substituted Hydroxyapatite Nanoparticles: An In Vitro Study

**DOI:** 10.7759/cureus.52177

**Published:** 2024-01-12

**Authors:** Swati Singh, Ravindra Kumar Jain

**Affiliations:** 1 Orthodontics and Dentofacial Orthopedics, Saveetha Dental College and Hospitals, Saveetha Institute of Medical and Technical Sciences, Saveetha University, Chennai, IND

**Keywords:** dental, orthodontic adhesives, antibacterial activity, nanoparticles, hydroxyapatite, cerium

## Abstract

Introduction

White spot lesions (WSLs) are early enamel caries lesions often seen in individuals receiving fixed orthodontic treatment. These lesions occur due to the buildup of plaque and the colonization of bacteria. WSL formation can be prevented by adequate oral hygiene measures and by the incorporation of antimicrobial nanoparticles (NPs) in orthodontic appliances and bonding systems. The aim of this research was to synthesize cerium-substituted hydroxyapatite nanoparticles (Ce-HAp NPs), characterize them, and assess their antimicrobial activity.

Materials and methods

This in vitro investigation involved the preparation of Ce-HAp NPs using the co-precipitation method, followed by their characterization using scanning electron microscope (SEM), energy-dispersive X-ray (EDAX), and Fourier transform infrared (FTIR). The NPs were prepared and subsequently added to an orthodontic adhesive. Antibacterial testing was conducted using the disc diffusion method against common oral pathogens (*Staphylococcus* *aureus*, *Lactobacillus acidophilus*, and *Streptococcus mutans*). The zones of inhibition were measured for two different concentrations of the adhesive.

Results

The Ce-HAp NPs were successfully prepared and had an irregular agglomerated shape, measuring 63 nm in size. The major characteristic chemical groups of Ce-HAp were PO_4_^3-^, OH-, and CO_3_^2-^, and it was confirmed by the FTIR spectrum. The EDAX results of the synthesized NPs showed theoretical weight percentages (Wt%) of O, 52.6%; Ca, 20.9%; P, 11.8%; C, 10.3%; and Ce, 4.3%. A higher concentration of 40 µg/mL (30 mm for *S. aureus* and *L. acidophilus *and 25 mm for *S. mutans*) showed good antibacterial activity against the tested bacterial strains, compared to control antibiotics.

Conclusion

Cerium oxide (CeO_2_)-HAp NPs were prepared and incorporated into an orthodontic adhesive. The prepared adhesive exhibited effective antibacterial activity against prevalent oral pathogens.

## Introduction

Nanoparticles (NPs) have been utilized in the biomedical field to combat bacterial drug resistance, thanks to their targeted antibacterial properties [1]. Numerous metal and metal oxide-based nanomaterials have been successfully researched for antibacterial purposes [2]. Nanomaterials play a significant role in physical and chemical processes due to their high surface-to-volume ratio [3]. These NPs have various benefits, including low cost, widespread commercial production, and use in the pharmaceutical industry [4,5]. In orthodontics, nanoparticles are used for coating brackets and incorporation in adhesives, mouthwashes, and toothpastes for antimicrobial effect [6]. Incorporating different NPs with antibacterial effects enhances the antibacterial and mechanical properties of various dental materials [7]. The substance should ideally reduce bacterial activity at the interphase of restoration. Hydroxyapatite (HAp) is a compatible biomaterial and is one of the main components of the bone and teeth with a structure that can be substituted to modify its physical, chemical, and biological properties [8,9]. HAp NPs have been previously used for remineralizing white spot lesions (WSLs), either individually or in combination with metal NPs [10]. Previously, orthodontic adhesives have been modified with silver (Ag) HAp NPs for the prevention of white spot lesions [11].

Cerium oxide (CeO_2_) NPs have bacteriostatic, bactericidal, and immunomodulating activity. Soluble Ce^3+^^ ^salts (nitrate, acetate, chloride, etc.) have long been employed previously for biomedical applications [12]. Cerium (Ce) itself has no biological importance in mammalian physiology. They display better antioxidant properties and a slower release of the metal than other metal oxide NPs [13]. The distinctive functional mechanism of cerium and cerium oxide-based nanomaterials allows for minimal toxicity and excellent antibacterial activity against pathogens. This mechanism involves the reversible change of the oxidation state between Ce(III) and Ce(IV) [14]. CeO_2 _has been used as a filler nanoparticle in restorative composite and reported to have a good antibacterial effect [15].

The structure of HAp can be easily substituted by other elements. Additionally, due to its numerous therapeutic benefits, cerium can be used as a substitute for calcium in HAp [16]. Since HAp NPs have been used for the remineralization of WSLs and cerium has demonstrated antibacterial properties, it is hypothesized that a combination of these two substances may be effective in the management of WSLs. Hence, the present study was attempted [17].

The aim of this study was to prepare CeO_2_-infused HAp NPs, characterize them, mix them in an orthodontic adhesive, and assess their antibacterial effect.

## Materials and methods

**Figure 1:**
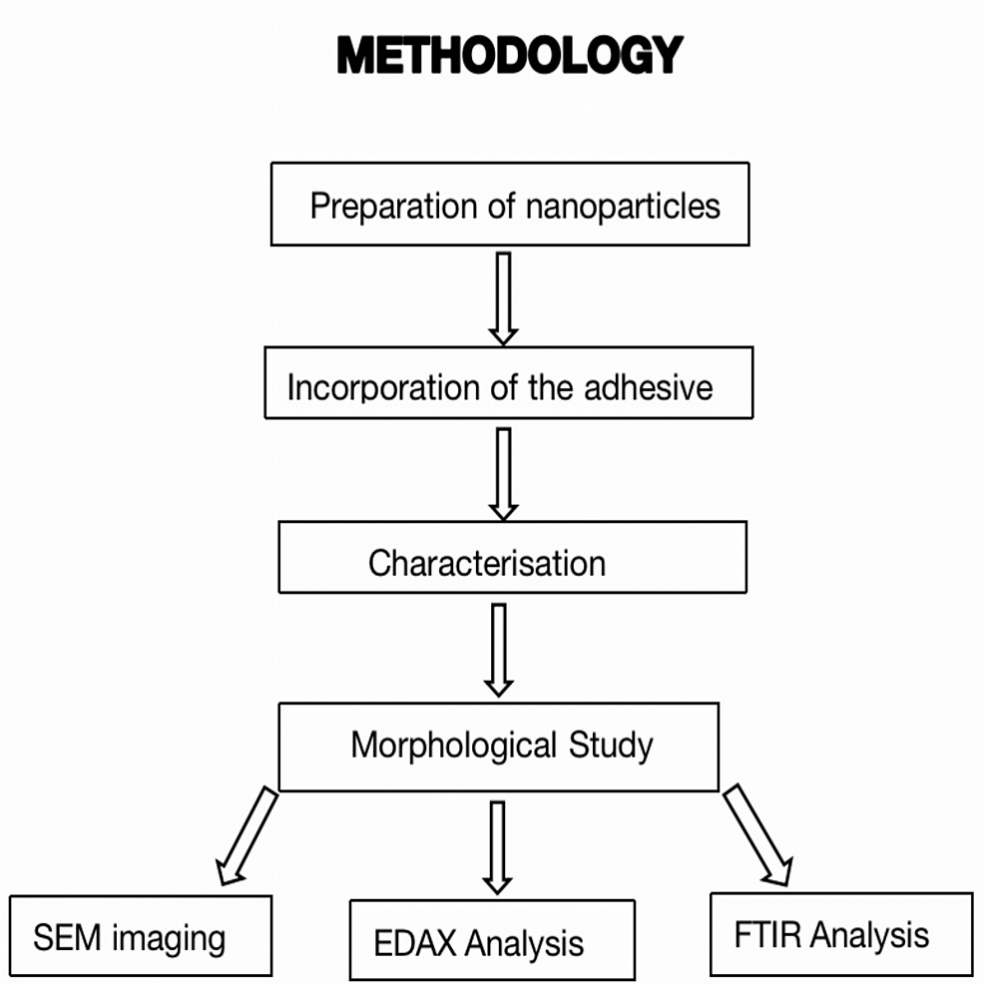
Flow diagram of the methodology SEM, scanning electron microscope; EDAX, energy-dispersive X-ray; FTIR, Fourier transform infrared

SRB/SDC/ORTHO-2103/22/079 was the Saveetha Review Board's authorization. Figure 1 shows the flow diagram of the methodology. 

Preparation of NPs

**Figure 2:**
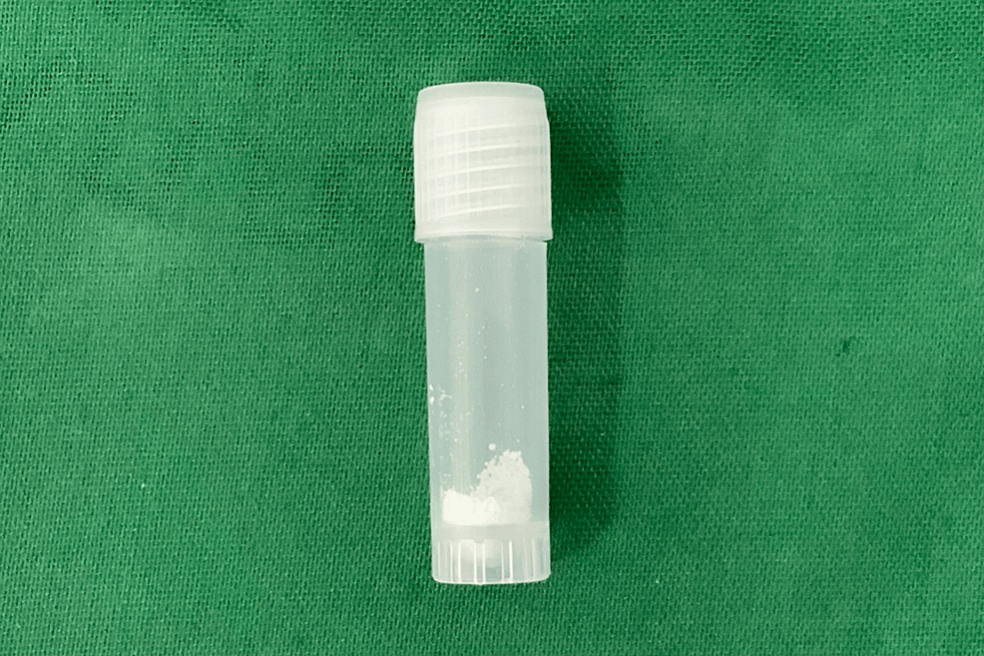
The obtained nanoparticle

The synthesis of Ce-substituted hydroxyapatite NPs by the co-precipitation method was performed in this study. To obtain Ce-substituted hydroxyapatite (Ce-HAp), a 1 M of Ce(NO_3_)_2_∙4H_2_O aqueous solution and a 0.02 M of cerium nitrate hexahydrate (Ce(NO_3_)_3_∙6H_2_O, Merck) aqueous solution were co-precipitated in a 0.6 M diammonium hydrogen phosphate (DAP) ((NH_4_)_2_HPO_4_) aqueous solution under continuous stirring for four hours at 100°C. After adding all solutions, the pH of the solution was maintained by adding NH_4_OH (Merck) to DAP. The pH of the solution was adjusted to 10.5 in the process. The prepared solution was stirred using a magnetic stirrer with heating at 90°C for eight hours in a fume hood cupboard. The gels were subsequently calcined for 1.5 hours at 750°C after being dried in an oven for 20 hours at 110°C. As a result, white granules were obtained (Figure 2) [16].

Incorporation in the adhesive

The Enlight orthodontic adhesive (Ormco Corporation, Orange, CA) was blended with an optimized weight percentage (Wt%) of Ce-substituted hydroxyapatite (HAp) NPs in a fume hood cupboard. Two grams of orthodontic adhesive was blended with 200 mg of Ce-substituted HAp NPs to attain a stoichiometric ratio of 10:1. This is because a higher weight percentage of NPs will impair proper dispersion in the adhesive [18]. As a result, one control group (without NPs) and one study group (orthodontic adhesive containing NPs) were prepared. For 10 minutes, a vortex machine (LabQuest Borosil, Pune, India) rotated at 600 rpm to combine NPs and the orthodontic adhesive. The prepared nanoparticle-containing adhesive was kept in previously washed and covered beakers to prevent water dispersion and exposure to light. After applying black Teflon tape to the beakers, they were sonicated for 60-90 minutes. Ice cubes were added to the water in the ultrasonicator system to maintain a consistent temperature of 0-5 degrees for the adhesive.

Characterization of Ce-substituted hydroxyapatite NPs

Following the synthesis, the NPs were characterized. The morphological study of the NPs and the admixed orthodontic adhesive was performed by scanning electron microscope (SEM) imaging and energy-dispersive X-ray (EDAX) spectroscopy analysis. The chemical studies were performed using Fourier transform infrared (FTIR) spectroscopy and energy-dispersive X-ray spectroscopy.

Morphological study

SEM imaging was used to evaluate the surface morphology of the nanocomposites (model: JSM-IT800, JEOL Ltd., Tokyo, Japan). The samples were mounted on an aluminum stub with carbon tape and then gold sputter-coated. They were observed under a SEM at 5 kV. The morphology of the Ce-HAp NPs was viewed at 0.5 μm magnification, and of the Ce-substituted HAp NPs, admixed adhesive was viewed at 1 μm magnification.

EDAX analysis was used to determine the chemical composition of the Ce-HAp NPs. The functional group analysis of the nanoparticles was studied using FTIR in the frequency range of 4000-400 cm^-1^ (Bruker ALPHA II, Billerica, MA).

Antibacterial activity

Gram-positive bacteria, *Staphylococcus aureus* (MTCC 740) and *Streptococcus mutans* (MTCC 890) and *Lactobacillus acidophilus* (ATCC 4356), were cultured in a nutrient broth flask. This flask contained all the essential nutrients to inhibit the growth of other microbes. The nanoparticle-incorporated Enlight orthodontic adhesive in two different concentrations (low concentration {LC} {20 µg/mL} and high concentration {HC} {40 µg/mL}) was cured, and six discs were used in this study. To prepare the composite discs, 5 mm-diameter circular metal molds were filled with the prepared adhesive and covered by glass slides. The adhesive discs were light-cured for 20 seconds using a light-emitting diode (LED) light source (Woodpecker, New Delhi, India); then, the adhesive discs were removed from the molds and sterilized. Standard antibiotics (amoxicillin and erythromycin) were used as controls to compare their effectiveness against both gram-positive and gram-negative bacteria. The samples were initially incubated for diffusion for 30 minutes at 4°C and then for bacterial activity for 24 hours at 37°C [19]. The test was considered successful when a zone of inhibition (ZOI) was observed around the well after the incubation period.

## Results

FTIR analysis

**Figure 3:**
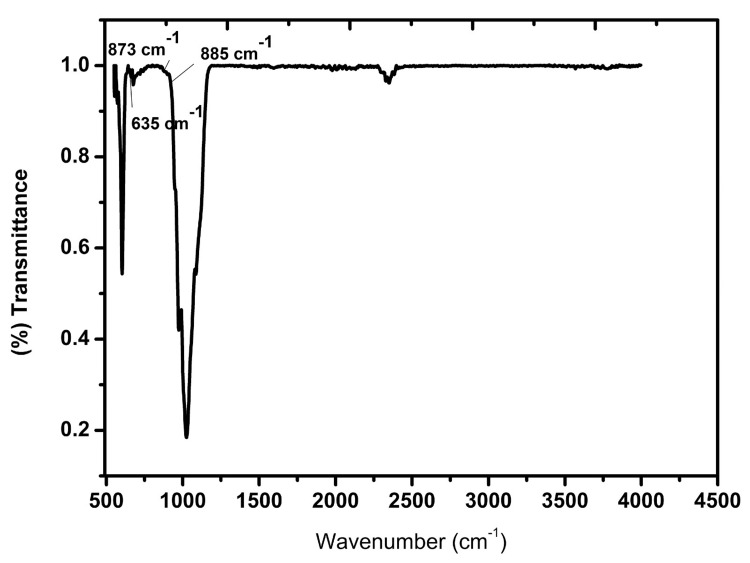
FTIR of Ce-containing HAp NPs FTIR, Fourier transform infrared; Ce, cerium; HAp NPs, hydroxyapatite nanoparticles

The major characteristic chemical groups of Ce-HAp were PO_4_^3-^, OH^-^, and CO_3_^2-^, as confirmed by the FTIR spectrum. The peak positions between 500-600 cm^-1 ^and 1000-1100 cm^-1^ were related to PO_4_^3-^ stretching vibrations. The low-intensity peak was observed at around 635 cm^-1^,^ ^corresponding to the OH^-^ bending vibration of water molecules. The characteristic peaks at 885 cm^-1 ^and 872 cm^-1^ are related to the CO_3_^2-^ substitution in the OH^-^ site in the Ce-HAp NPs and the interaction between the CO_3_^2-^ and Ce(IV) (Figure 3). The observed peaks of Ce-substituted hydroxyapatite NPs are almost similar to that of pure hydroxyapatite due to the less amount (Wt%) of Ce doping with HAp (2 g of orthodontic adhesive blended with 200 mg of Ce-substituted HAp NPs). This less incorporation of Ce does not change the crystal structure of pure HAp. The CO_3_^2-^ ion interaction may be due to the synthetic method followed for the preparation of HAp nanoparticles.

EDAX analysis

The main elements of existence from Ce-HAp were identified in the elemental distribution as shown in the EDAX spectrum. The difference in the intensities of the O, Ca, P, and C elements in the EDAX spectrum and the height of the peak intensity confirm the presence of the (Wt%) elemental distribution. The presence of the above elements in the EDAX spectrum is related to pure HAp. The presence of the Ce element in the spectrum confirmed the formation of Ce-substituted hydroxyapatite nanoparticles. The observed results led to the conclusion that no other impurities were formed during the synthesis process. The chemical composition of CeO_2_-HAp NPs was analyzed using EDAX, and the results are shown in Table 1. The theoretical weight percentages of O, Ca, P, C, and Ce in the sample were 52.6%, 20.9%, 11.8%, 10.3%, and 4.3%.

**Table 1:** EDAX of cerium (Ce)-substituted hydroxyapatite nanoparticles Wt%, weight percentage; EDAX, energy-dispersive X-ray

	Wt%	σ (atomic percentage)
O	52.6	0.5
Ca	20.9	0.2
P	11.8	0.1
C	10.3	0.6
Ce	4.3	0.1

SEM analysis

**Figure 4:**
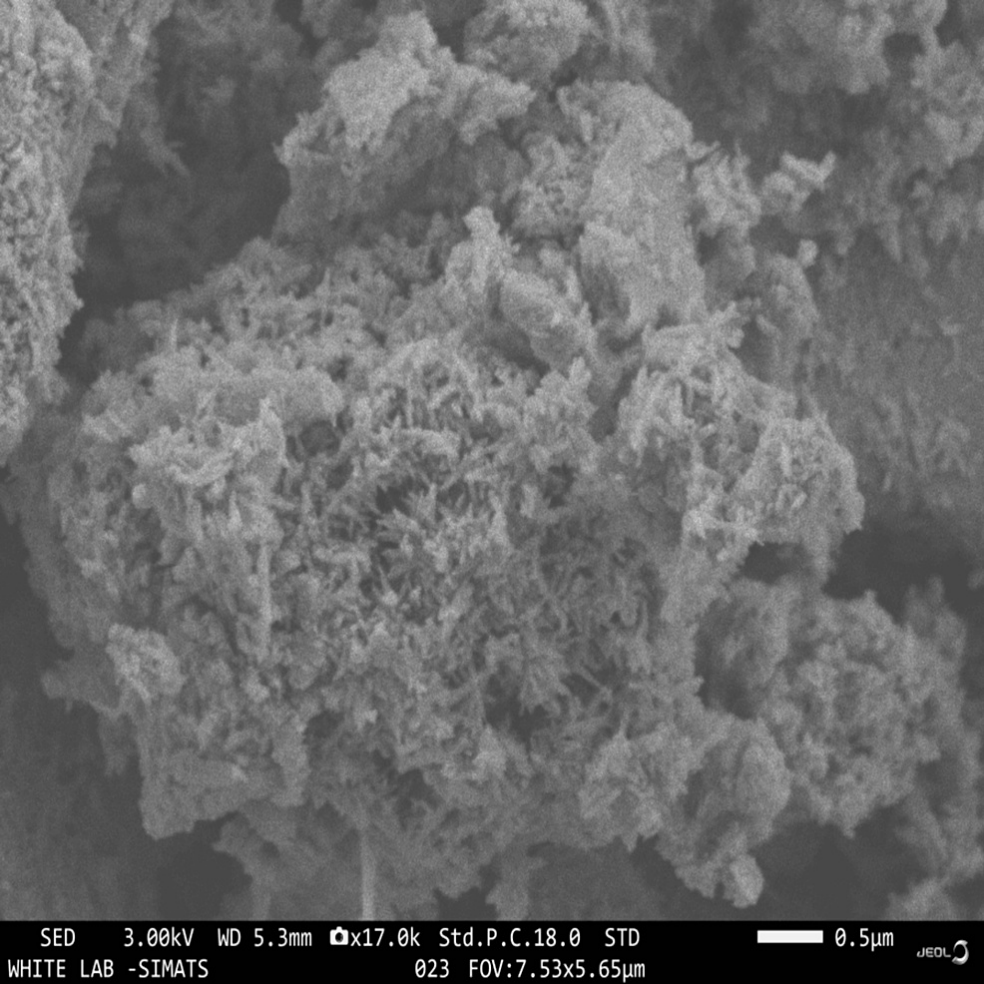
SEM image of prepared Ce-HAp NPs SEM, scanning electron microscope; Ce-HAp NPs, cerium-substituted hydroxyapatite nanoparticles

Structural differences and morphology variations were observed for the cerium-substituted hydroxyapatite and adhesive-containing Ce-HAp nanoparticles. From SEM images, it could be noted that the introduction of the orthodontic adhesive to the Ce-HAp slightly influences the morphology of the overall adhesive. This may probably be due to the ionic replacements in the crystal lattice of pure HAp to compensate for the charge imbalance between Ce ions and the adhesive. Island and rough surface morphology were observed for Ce-substituted HAp (Figure 4).

**Figure 5:**
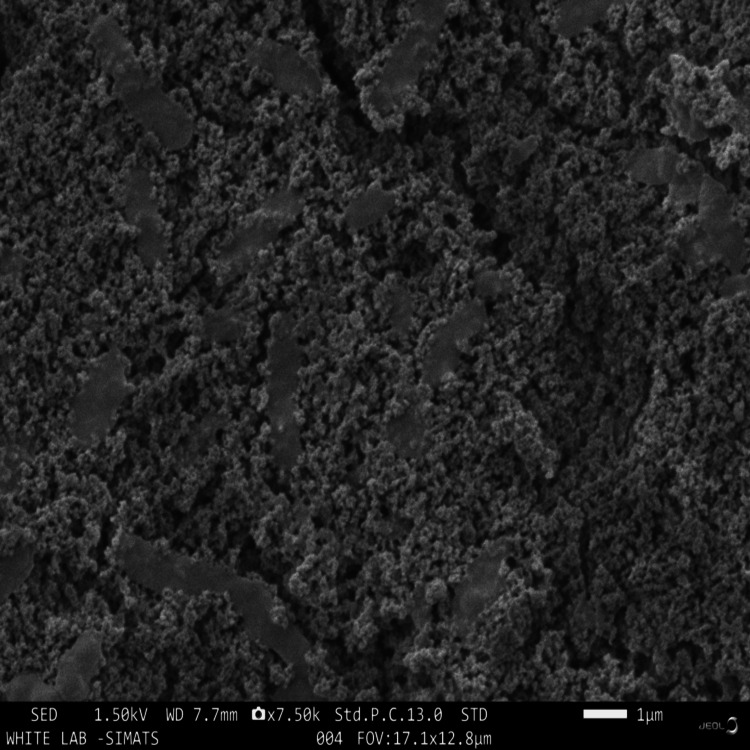
SEM image of Ce-substituted HAp NP admixed adhesive SEM, scanning electron microscope; Ce, cerium; HAp NP, hydroxyapatite nanoparticle

Ce-substituted hydroxyapatite nanoceramics, when mixed with orthodontic adhesive, exhibited a rough surface morphology characterized by the presence of Ce-substituted HAp dispersed within the orthodontic adhesive (Figure 5). The size of the Ce-substituted HAp was found to be 59 nm using the SEM image in the ImageJ software (National Institutes of Health, Bethesda, MD).

Antibacterial activity

**Figure 6:**
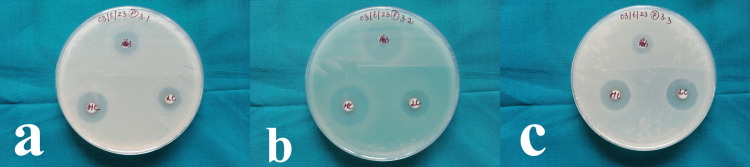
Zones of inhibition against antibiotics at different concentrations a) *Staphylococcus aureus*, b) *S. mutans*, and c) *L. acidophilus*

The antibacterial behavior of orthodontic adhesive containing Ce-substituted HAp NPs against the microbial pathogens *S. mutans*, *E. coli*, and *S. aureus* at concentrations of 20 µg/mL and 40 µg/mL is given in Figure 6. Zone of inhibition (ZOI) results are presented in Table 2.

**Table 2:** Zone of inhibition in millimeters of the Ce-substituted HAp NP adhesive in two different concentrations Ce, cerium; HAp NP, hydroxyapatite nanoparticle

Bacteria	Control	Low concentration (20 µg)	High concentration (40 µg)
Staphylococcus aureus	20 mm	28 mm	30 mm
Streptococcus mutans	29 mm	14 mm	25 mm
Lactobacillus acidophilus	20 mm	28 mm	30 mm

The antibacterial activity was higher in the 40 µg/mL concentration than in the control and low concentrations (20 µg/mL). This significant antimicrobial activity of the orthodontic adhesive is due to the presence of Ce ions and HAp (Figure 6).

## Discussion

Enamel demineralization around brackets, often known as "white spot lesions," is a very complicated issue that occurs during orthodontic treatment. It is caused by inadequate oral hygiene and an increase in oral microflora [20]. Orthodontic adhesives, which have a polymer matrix, can accumulate microbes that form a supragingival biofilm. This biofilm can harbor bacteria such as *S. mutans*, which are known to play a role in the development of dental caries and white spot lesions (WSLs). *Streptococcus mutans* colonizes and binds to bonding agents, thereby increasing the prevalence of enamel demineralization [21].

Orthodontic adhesives can be enhanced by adding certain nanoparticles, which can improve their antibacterial resistance and shear bond strength. Previous studies have reported the testing of various NPs after incorporating them into orthodontic adhesives as a strategy for managing WSLs [22,23]. Titanium dioxide nanoparticle-modified orthodontic adhesives exhibit a significant antibacterial effect [18]. Similarly, other studies have reported testing Ag, ZnO, and polyethylenimine nanoparticles into composite resins and reported positive results [23]. In the present study, Ce-substituted HAp NPs were used. HAp NPs have been found to effectively remineralize enamel, and when Ce is incorporated into orthodontic adhesive separately, it has shown significant antimicrobial activity against oral microbes [24,25]. The mechanism by which CeO_2 _nanoparticles exert their antibiotic effects is through their adsorption onto the negatively charged bacterial membranes. Nanoparticles adhere to the surface of bacteria and can penetrate through the bacterial membrane barrier, affecting the transportation processes between the bacterial cell and fluid. This interaction also hinders bacterial growth [15]. To the best of our knowledge, no study has been conducted in the past that has utilized Ce with HAp NPs and incorporated them into orthodontic adhesives.

In the present study, we successfully prepared Ce-infused HAp NPs and characterized them using FTIR. We confirmed the formation of nanoparticles. The morphology of the NPs was confirmed using SEM, revealing irregular agglomerated structures with a mean diameter of 59 nm when measured with the ImageJ software. The elemental composition was verified with EDAX analysis. Previous studies have reported on the synthesis of HAp NPs by precipitation, sol-gel, pyrolysis, and microwave methods [16] and Ce NPs using sonochemical method [25], modified hydrothermal method [26], and co-precipitation methods [15]. In the present study, the co-precipitation method was used for the synthesis of Ce-HAp NPs. In a previous study, the preparation of Ce-substituted HAp NPs was conducted using a sonochemical method. The NPs were characterized using FTIR, and the elemental composition was determined using EDAX, which is similar to the approach employed in the present study [27,28]. Upon analyzing the antibacterial effects of the NP-infused adhesive, it was observed that the high-concentration NP-containing adhesive (40 µg/mL) exhibited significant antibacterial activity against the tested bacteria, surpassing that of the control antibiotic. In the study conducted by Poosti et al. [18], an orthodontic adhesive substituted with cerium oxide nanoparticles (CeO_2_ NPs) was prepared. The researchers evaluated its antibacterial activity against *S. mutans* using a disc diffusion test and also performed an assessment of its antibiofilm activity. It was concluded that the CeO_2 _NP-containing adhesive exhibited effective antimicrobial and antibiofilm activity against *S. mutans*. In a previous study by Varghese et al. [15], a novel restorative composite based on CeO_2_ nanofiller was prepared. The study observed significant antimicrobial activity against *Streptococcus* and *Lactobacillus* species.

Further research is needed to investigate the cytotoxicity and biocompatibility of compounds that contain cerium and cerium oxide.

Limitations

One limitation of this study is that it involves an in vitro analysis. Being an in vitro study is a limitation in itself, as in vivo studies closely relate to the clinical situation. Additional animal studies and long-term effect monitoring are expected to improve the utilization of cerium-related and cerium oxide-related antibacterial compounds. Further research is needed to investigate the cytotoxicity and mechanisms of novel compounds based on cerium and cerium oxide.

## Conclusions

Ce-substituted hydroxyapatite NPs were successfully synthesized by the co-precipitation method and, when mixed with orthodontic adhesive, exhibited a rough surface morphology. Chemically synthesized Ce-HAp NPs in orthodontic adhesive demonstrated a good antibacterial effect, and the antibacterial activity was higher in the 40 µg/mL concentration than in the control and low concentrations. The significant antimicrobial activity of the orthodontic adhesive is due to the presence of Ce ions, which had substituted HAp NPs. It can be concluded that Ce-substituted HAp NPs can be incorporated with orthodontic adhesives to impart an antibacterial effect, thereby preventing enamel demineralization or white spot lesions around orthodontic brackets.
